# T1, T2 and T2* mapping following acute myocardial infarction in a porcine model: Regional and serial alterations during infarct healing

**DOI:** 10.1186/1532-429X-15-S1-P86

**Published:** 2013-01-30

**Authors:** Nilesh R Ghugre, Yuesong Yang, Xiuling Qi, Jennifer Barry, Venkat Ramanan, Jeff A Stainsby, John J Graham, Kim A Connelly, Graham Wright

**Affiliations:** 1 Physical Sciences, Sunnybrook Research Institute, Toronto, ON, Canada; 2 Medical Biophysics, University of Toronto, Toronto, ON, Canada; 3 Cardiology, St. Michaels Hospital, Toronto, ON, Canada; 4 Applied Sciences Laboratory, GE Healthcare, Toronto, ON, Canada

## Background

Quantitative T1 and T2 cardiovascular magnetic resonance (CMR) approaches have been utilized to detect edema post acute myocardial infarction (AMI). However, a systematic comparison between the two measures is currently lacking. Furthermore, these relaxation measurements may also be confounded by opposing effects of intramyocardial hemorrhage that is typically not taken into account. The purpose of our study was to compare regional and serial fluctuations in T1 and T2 post-AMI and additionally quantify T2* that is a more specific marker of hemorrhage.

## Methods

A porcine model of myocardial infarction was employed involving a 90 min LAD occlusion followed by reperfusion. Pigs (N=3) were imaged on a 1.5T scanner (GE Healthcare) in a healthy state (control) and at day 2 and week 4 post-AMI. The following sequences were utilized for quantification: T2 with multi-echo fast-spin-echo (4 echo's; TE=5.6-105 ms); apparent T1 by modified look-locker with saturation recovery (TI=50-3000 ms); T2* by gradient echo (2 echo's; TE=1.4, 15 ms; TR=18ms). Measurements were reported in two regions-of-interest (ROI): 1) infarct zone defined from late gadolinium enhancement images; and 2) remote uninfarcted myocardium. Two short-axis slices were analyzed for each study; the anatomical location was identical for all three sequences.

## Results

**Figure 1:**
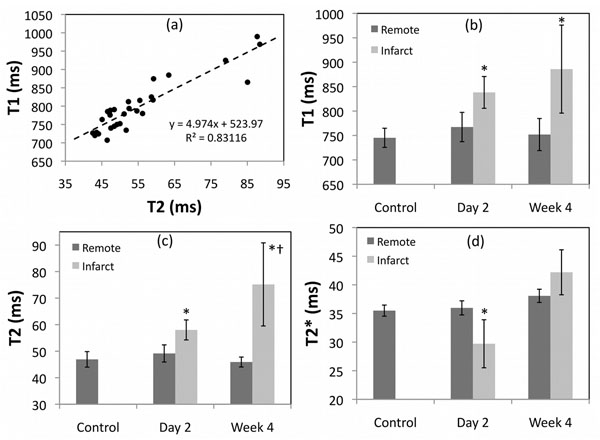
Linear correlation between T1 and T2 is shown in (a). The serial evolution of T1, T2 and T2* is demonstrated in infarct and remote myocardium as shown in (b), (c) and (d), respectively. Error bars represent standard deviation. *p<0.05, compared to control; †p<0.05, compared to day 2.

Fig. 1 a shows the linear regression between T1 and T2 values when taken across all ROI's and time points. Both T1 and T2 were significantly elevated at day 2 (p=0.0003, p=0.0001) and week 4 (p=0.01, p=0.005) post-AMI compared to control, respectively; see Fig. 1b and 1c. Reduced T2* at day 2 (p=0.05) suggested the presence of hemorrhage in all animals; see Fig. 1d. Percent change in T2 (relative to control) was significantly greater than that for T1 at both day 2 (25±12 vs. 13±3 %, p=0.05) and week 4 (62±36 vs. 20±13 %, p=0.03). A lower apparent change at day 2 may be suggestive of influence from hemorrhage on both T1 and T2.

## Conclusions

T1 and T2 are highly correlated in AMI; however, T2 is a more sensitive parameter to detect the underlying alterations following AMI. In the early sub-acute phase, both T1 and T2 may be affected by counteracting effects of edema and hemorrhage, unlike T2*. Furthermore, in the late phase when hemorrhage has resolved, sensitivity of T2 towards edema appears to be greater in comparison to T1; T1 may possibly be reduced due to chronic remodeling. Simultaneous characterization of these relaxation parameters may potentially offer useful insights into the complex remodeling processes that follow AMI.

## Funding

We would like to acknowledge funding support from the Ontario Research Fund, the Canadian Institutes of Health Research and GE Healthcare.

